# From form to function: m^6^A methylation links mRNA structure to metabolism

**DOI:** 10.1016/j.jbior.2022.100926

**Published:** 2023-01

**Authors:** Braulio Martinez De La Cruz, Marousa Darsinou, Antonella Riccio

**Affiliations:** UCL Laboratory for Molecular Cell Biology - University College London, Gower Street, WC1E 6BT, London, UK

## Abstract

Reversible N6-methyladenosine (m^6^A) RNA modification is a posttranscriptional epigenetic modification of the RNA that regulates many key aspects of RNA metabolism and function. In this review, we highlight major recent advances in the field, with special emphasis on the potential link between m^6^A modifications and RNA structure. We will also discuss the role of RNA methylation of neuronal transcripts, and the emerging evidence of a potential role in RNA transport and local translation in dendrites and axons of transcripts involved in synaptic functions and axon growth.

## 1 Introduction

The discovery of a reversible N6-methyladenosine (m^6^A) RNA modification, along with advances in sequencing, have revealed a critical new layer of post-transcriptional messenger RNA (mRNA) regulation. m^6^A is the most common endogenous mRNA modification with each mRNA molecule having on average 2 modifications (Meyer et al., 2012; Dominissini et al., 2012). The characterisation of m^6^A-binding proteins – which include a writer complex that adds the modification to mRNA (Liu et al., 2013; Zhang et al., 2022a; Růžička et al., 2017), erasers to remove them (Jia et al., 2011; Zheng et al., 2013), and readers that are effector proteins (Li et al., 2017a; Xiao et al., 2016a; Wojtas et al., 2017; Theler et al., 2014; Zaccara et al., 2019a) – capable of changing mRNAs post-transcriptionally opened an exciting new field of epitranscriptomics focused on the dynamic m^6^A modifications and the role in RNA metabolism and gene expression (Meyer and Jaffrey, 2014a; Dominissini, 1979).

A major question surrounding m^6^A RNA modifications is centred on specificity. Early sequencing studies mapping m^6^A modifications to the transcriptome found a clear but malleable motif with the modified adenosine always located next to a cytosine (Meyer et al., 2012; Dominissini et al., 2012; Martinez De La Cruz et al., 2021). The DRACH motif (D = A, G, or U; R = A/G, H = A, C, or U) however, is not a rare sequence in mRNAs, and most DRACHs are not methylated (Meyer et al., 2012; Dominissini et al., 2012). Instead, it is now believed that RNA secondary structure plays a key role in determining m^6^A RNA modifications. This is because more disordered or loose mRNA regions are more accessible to the writer METTL3 methyltransferase complex and therefore more likely to be methylated (Choe et al., 2018; Guo and Shorter, 2015). Thus, in addition to the RNA sequence, the context surrounding it is equally important in determining the methylation status.

In the past decade, the functional effects of RNA methylation have also been extensively investigated. m^6^A plays significant roles in cellular physiology through a carefully orchestrated binding of writers, erasers, and readers to m^6^A at different times (Bodi et al., 2012; Zhou et al., 2015; Haussmann et al., 2016; Lence et al., 2016; Cui et al., 2017a; Zhang et al., 2017, 2022b; Ma et al., 2019; Lin et al., 2016; Han et al., 2019; Heck et al., 2020; Zhao et al., 2021). In *Arabidopsis* for example, restriction of MTA m^6^A writer (a METTL3 homolog) expression past the embryonic stage, leads to severe developmental defects (Bodi et al., 2012). Heat-shock also causes an increase of m^6^A-methylated mRNA in yeasts, followed shortly by increased expression of YTH Domain Family 2 (YTHDF2) protein (Zhou et al., 2015). In *Drosophila melanogaster*, m^6^A strongly influences sex determination by inducing alternative splicing of the female-determining *Sxl* transcript, in a process regulated by the m^6^A reader YT521-B (Haussmann et al., 2016; Lence et al., 2016). Many cancer cell lines and tumours globally overexpress modified RNA (Cui et al., 2017a; Zhang et al., 2017; Ma et al., 2019; Lin et al., 2016) and in *Ythdf1*^*−/−*^ mice, tumour neoantigen cross-presentation is reduced resulting in impaired immune evasion (Han et al., 2019). In human iPSCs, m^6^A levels are increased in pluripotent proliferating cultures but are quickly downregulated during neural differentiation through YTHDF2-mediated degradation (Heck et al., 2020). Latest research has shown that m^6^A-modified RNA levels are changed in the brain of patients with dementia, although a direct relationship between m^6^A-modified RNA and the mechanisms of neurodegeneration remains unclear (Zhang et al., 2022b; Zhao et al., 2021). These examples demonstrate that m^6^A-modified mRNAs play a crucial role both at cellular and organismal levels. However, we still do not have a clear picture regarding how structural and physical changes associated with modified mRNAs affect these functions.

In this review, we highlight the latest research that provides insights into how m^6^A modifications affect the secondary structure and translatability of mRNA, as well as the RNA-binding proteins (RBPs) that interact with RNA to build transient molecular scaffolds necessary for RNA modifications and metabolism. Instances in which data are still not available will also be mentioned. Finally, we will detail evidence supporting the hypothesis that RNA modifications play a pivotal role in regulating mRNA transport, translation, and degradation in the nervous system.

## 2 m^6^A-mediated RNA metabolism

As for many epigenetic modifications, m^6^A is regulated by three groups of proteins. Writers, which add the modification to mRNA, are found in the nucleus and therefore fall outside of the scope of this review. For more information on writers, we direct the reader to two recent reviews by He and Meyer (He and He, 2021; Meyer and Jaffrey, 2017). m^6^A modifications can be bound by multiple readers that specifically regulate mRNA processing. One of the most important questions in the field is why seemingly redundant readers such as YTHDF1/2/3 show distinct protein expression profiles and respond to different stimuli. In addition to YTHFDs, other proteins are capable of reading m^6^A modifications directly or indirectly, as will be discussed below. The presence of m^6^A readers in the cytoplasm allows the interaction with m^6^A modified RNAs, contributing in mRNA transport, translation and degradation (Zaccara et al., 2019a).

### 2.1 mRNA translation

**Fig. 1:**
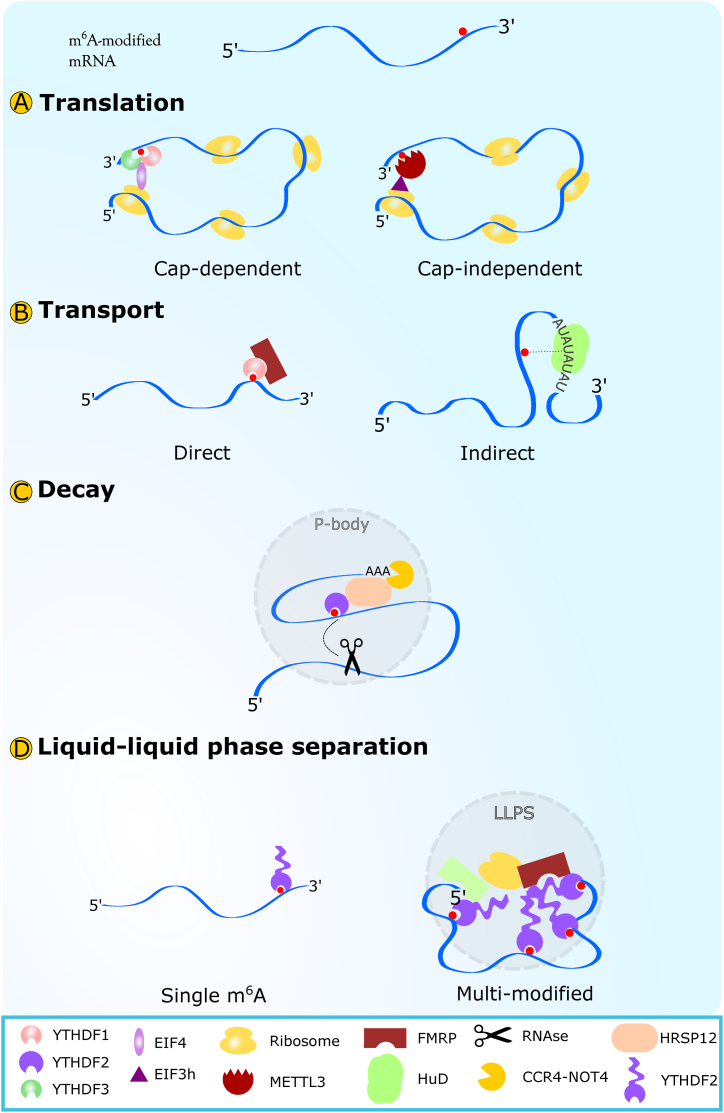
Potential structural changes related to m^6^A mRNA modifications leading to Translation (**A**), Transport (**B**), Decay (**C**) and liquid-liquid phase separation (**D**).

The YTH Domain Family is the main family of cytoplasmic m^6^A readers and comprises YTHDF1, YTHDF2, and YTHDF3. YTHDF1 plays a crucial role in promoting translation of m^6^A-modified RNAs by interacting with initiation factors and allowing ribosome loading (Fig. 1A) (Meyer and Jaffrey, 2014a; Wang et al., 2015). The activity is further enhanced by the interaction of YTHDF1 with YTHDF3. A combination of pull-down assays, **P**hoto**A**ctivatable **R**ibonucleoside-enhanced **C**ross-**L**inking and **I**mmuno**P**recipitation (PAR-CLIP), and ribosome profiling, indicated that YTHDF1 and YTHDF3 both bind to ribosomal subunit proteins with high efficiency (>70% of 40 S and 60 S proteins). Knockdown of either protein severely reduces the translational efficiency of their common targets, but knockdown of YTHDF3 only does not affect the translation of its unique binding targets, perhaps suggesting a different role of YTHDF3 in the absence of functional interaction with other readers (Li et al., 2017a; Shi et al., 2017). At the RNA level, the working model for common targets entails that after anchoring to m^6^A modifications around the stop codon, YTHDF1 and YTHDF3 bind to initiation factors, and both proteins recruit ribosomal subunits (Fig. 1A). Following the interaction with YTHDF1 and YTHDF3, the RNA is bent forming a secondary structure that allows the scanning of the mRNA by the complex while building multiple ribosomes on it (Fig. 1A) (Li et al., 2017a).

In a similar but less efficient mechanism, m^6^A modifications within the 5′ untranslated region (5′UTR) of mRNAs can be translated independently of the 5′ cap (Fig. 1A). Initially, it was found that in HeLa cell lysates and 293 T cells, a single m^6^A modification in the 5′UTR of transcripts would allow translation without the cap-dependent eIF4 complex (Meyer et al., 2015a). Further experiments showed that eIF3 binds directly to the m^6^A in the 5′ UTRs, thereby acting as an m^6^A reader. Most eIF3-binding sites were located on m^6^A consensus motifs (Meyer et al., 2015a), and the interaction resulted in cap-independent recruitment of the 43 S preinitiation complex (Wang et al., 2015). In long-lived endocrine mutant mice, pharmaceutical inhibition of cap-dependent translation resulted in increased translation of DNA repair and mitochondrial stress proteins, without changes of mRNA levels (Ozkurede et al., 2019). Similarly, in human and mouse cells, upon heat shock or other metabolic and physical stresses the number of 5′ UTR-methylated mRNAs (many of them oncogenic, based on MeRIP-seq gene ontology analysis), and the translation increased despite no apparent change of mRNA levels (Choe et al., 2018; Meyer et al., 2015b). A few recent studies using a combination of **R**NA **I**mmuno**P**recipitation (RIP), mass spectrometry, and electron microscopy suggested that 5′ cap-independent translation is mediated by an alternative function of the m^6^A writer protein METTL3. During m^6^A addition to the mRNA, METTL3 binds to the eIF3h subunit and circularises the mRNA molecule to bring eIF3 closer to the translation initiation site, allowing the complex to recruit both YTHDF proteins and ribosomes (Fig. 1A) (Choe et al., 2018; Sinha et al., 2021). Thus, both mechanisms of m^6^A-mediated translation involve the bending or circularisation of mRNA in a 3D space.

Given that large RBP complexes bind to mRNA during translation (Hentze et al., 2018), these structural discoveries provide a model supporting the hypothesis that the anchoring of proteins to m^6^A affect their functions. Future studies will help to shed light on whether multiple concomitant mRNA modifications may determine the circularisation of m^6^A-mRNA by regulating RNA-protein interaction.

### 2.2 mRNA transport

An additional role of m^6^A readers observed in various cell types is related to the cytoplasmic transport of methylated mRNA (Fig. 1B) (Meyer and Jaffrey, 2017; Zaccara et al., 2019b; Patil et al., 2018). Many well-characterised RBPs associated with mRNA transport to distal cytoplasmic sites in neurons (Majumder et al., 2017; Ascano et al., 2012; Wächter et al., 2013; Fallini et al., 2011), including FMRP, IGFBP1/2/3, and HuD (Dominissini et al., 2012; Huang et al., 2018; Wang et al., 2014) interact with mRNA in a m^6^A dependent manner. Reduction of global m^6^A levels decreases protein binding to their targets. Inversely, knockout of FMRP, IGFBP1/2/3, and HuD results in decreased cytoplasmic translocation of m^6^A-modified transcripts (Huang et al., 2018; Zhang et al., 2018; Edens et al., 2019).

At a structural level, m^6^A readers can be further subdivided into two groups depending on the mechanism by which they regulate mRNA transport. The first mechanism is that in addition to directly binding to m^6^A modifications (Huang et al., 2018; Arguello et al., 2017; Ren et al., 2021; Edupuganti et al., 2017), FMRP and IGFBP2 proteins also interact with YTH proteins, as observed in the developing nervous system in *Drosophila* and in 293 T cells with bioID proximity mapping of the YTHDF interactome (Youn et al., 2018a; Worpenberg et al., 2021). This suggests that similarly to what observed for RNA translation, YTHDF proteins may serve as a scaffolding that links FMRP and IGFBP2 to m^6^A-modified mRNAs (Fig. 1B). A recent study in hippocampal neurons found that knockdown of YTHDF2 and YTHDF3 led to a significant reduction of *Camk2a* and *Map2* transcripts localisation to neurites (Flamand and Meyer, 2022), an effect that may be attributable to impaired recruiting of FMRP or IGF2BP. Thus, despite the often-reported redundancy of YTHDF proteins based on their binding to m^6^A (Lasman et al., 2020; Kontur et al., 2020; Li et al., 2020), their affinity for distinct RBPs may result in specific physiological functions.

The second mechanism relies on the increased accessibility to binding sites provided by m^6^A switch (Liu et al., 2015). m^6^A switch refers to the destabilisation of m^6^A-U base pairs in RNA loops which leads to a partial linearization of RNA and increased RBPs binding (Roost et al., 2015). In the case of HuD and other ELAV-like proteins, the reported RNA binding site does not contain a m^6^A consensus sequences but the site has an AU-rich region (Park et al., 2000). Such regions may form stem loops amenable to m^6^A switches (Liu et al., 2015) and it is possible that even though HuD does not bind directly to m^6^A, methylation may be still necessary for its recruitment to RNA (Fig. 1B).

### 2.3 mRNA decay and stability

YTHDF2 is the main m^6^A reader responsible for mRNA decay. YTHDF2 shows the strongest association with proteins in processing bodies (Youn et al., 2018b), which are well-characterised sites of mRNA degradation (Kulkarni et al., 2010). Similar to translation and translocation, YTHDF proteins can also mediate degradation by recruiting other specialised partners. For example, YTHDF2 promotes the endoribonucleolytic cleavage of both mRNA and circular RNAs by associating with the RNAse P/MRP complex (Fig. 1C) (Park et al., 2019). In this process, YTHDF2 binds to m^6^A at the 3′UTR, although the functional outcome is determined by whether a HRSP12 protein binding site is located upstream of the m^6^A modification. If HRSP12 is recruited, the RNAse P/MRP complex degrades the mRNA by cleavage. It should be noted that the upstream site may vary, and it is likely dependent on RNA tertiary structure. This requires that mRNA must fold forming a structure that is accessible to RNAseP, resulting in partial circularisation of the RNA molecule (Park et al., 2019). In the absence of an HRSP12 adaptor, YTHDF2 directly binds to the CCR4-NOT complex (Fig. 1C), which mediates RNA decay through 3′ end cleavage of the polyA tail (Boland et al., 2013; Du et al., 2016).

RBPs termed anti-readers also recognise the secondary structure of m^6^A-mRNA, and they are repelled by it (Arguello et al., 2017). Anti-readers include G3BP proteins and LIN28A (Edupuganti et al., 2017). G3BP RNA binding sites closely overlap with m^6^A motifs, and binding of G3BP to RNA is dependent on a lack of methylation (Edupuganti et al., 2017). However, even in cases where G3BP bound to alternative motifs, the same repellent effect was observed, perhaps through changes of RNA loops due to m^6^A switches (Edupuganti et al., 2017). The half-life of RNA unmethylated and bound to G3BP was significantly longer, and m^6^A methylation correlated with decreased half-life (Edupuganti et al., 2017) possibly through the decay processes described above.

### 2.4 The role of m^6^A in liquid-liquid phase separation

Liquid-liquid phase separation (LLPS) is a biological phenomenon by which components of similar hydrophobic characteristics accumulate and form highly concentrated dynamic condensates, such as granules and membrane-less intracellular structures. Stress granules, ribonucleoprotein complexes, and processing bodies in neurons are all found within LLPS (Garcia-Jove Navarro et al., 2019), and in this state, higher contact dwell times result in higher metabolic activity (Case et al., 2019).

Given that LLPS condensates are important sites of RNA metabolism, the role of m^6^A on phase separation has been investigated. In cell lines, multi-modified, but not singly-modified mRNAs promote phase separation when bound to YTHDF proteins (Fig. 1D) (Gao et al., 2019; Ries et al., 2019). Furthermore, FMRP and G3BP undergo a phase-switch depending on the methylation status of target mRNAs, thereby promoting stress granule formation (Fu and Zhuang, 2020; Zhang et al., 2022c). The biophysical basis of this phenomenon derives from the unique properties of YTHDF proteins. They contain a 15 kDa YTH domain that forms a hydrophobic cage with m^6^A modified RNAs (Xu et al., 2014; Luo and Tong, 2014). The remaining ∼40 kDa are composed of a P/Q/N-rich low-complexity, hydrophobic domain. Accumulation of YTHDF proteins strongly bound to methylated mRNA induces LLPS and forms a hydrophobic structure containing mRNAs bound by YTH domains on the outside, and low-complexity domains aggregating on the inside (Fig. 1D). In this model, the low-complexity domains of YTH proteins remain accessible to serve as a scaffold for other m^6^A readers, ribosomes, or granule components that may contribute to mRNA processing (Fig. 1D), therefore complementing previous models of m^6^A-RNA metabolism.

## 3 m^6^A modifications of neuronal transcripts

Initial sequencing studies performed in the mouse brain revealed a high enrichment of methylated RNA in the brain and an accumulation of m^6^A around the stop codon and the 3′ UTR of neuronal transcripts (Meyer et al., 2012; Dominissini et al., 2012). More recently, sequencing in human brain, found a similar m^6^A distribution, with white matter tissue containing a higher proportion of m^6^A sites within the 3′ UTR compared to other brain regions (Martinez De La Cruz et al., 2021). The distribution of m^6^A is especially interesting given that neurons express alternative polyadenylated mRNA isoforms with the longest 3’ UTRs and contain some of the best-characterised localisation elements (Andreassi and Riccio, 2009). Importantly, alterations of m^6^A expression have been linked to dementia (Han et al., 2020; Huang et al., 2020). One limitation of these studies is that although many new sequencing techniques targeting m^6^A have been developed, the extremely low amount of RNA that can be isolated from neuronal subcellular compartments has so far prevented high-depth m^6^A sequencing of transcripts transported in dendrites, for example (See Box 1 and Table 1).

**Table 1:**
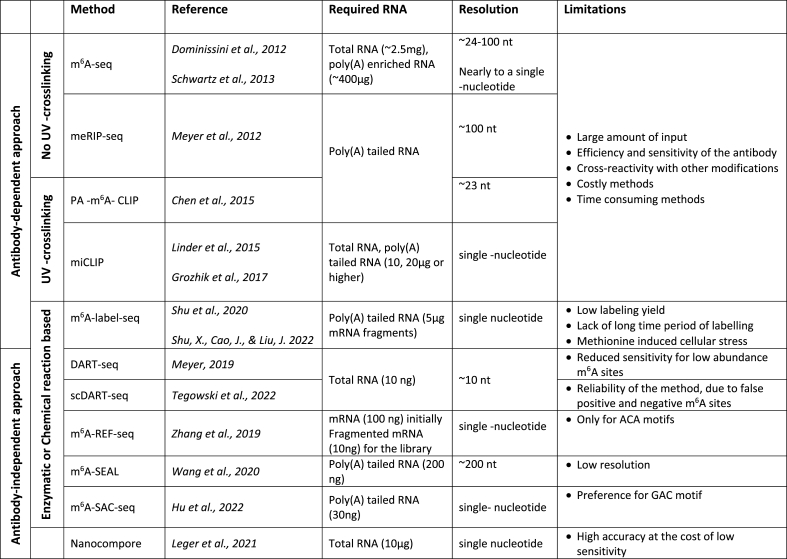
Methods used to detect m^6^A modifications (Dominissini et al., 2012; Schwartz et al., 2013; Meyer et al., 2012; Chen et al., 2015; Linder et al., 2015; Grozhik et al., 2017; Shu et al., 2020; Shu et al., 2022; Tegowski et al., 2022; Zhang et al., 2019; Wang et al., 2020; Hu et al., 2022; Leger et al., 2021).

**Fig. 2:**
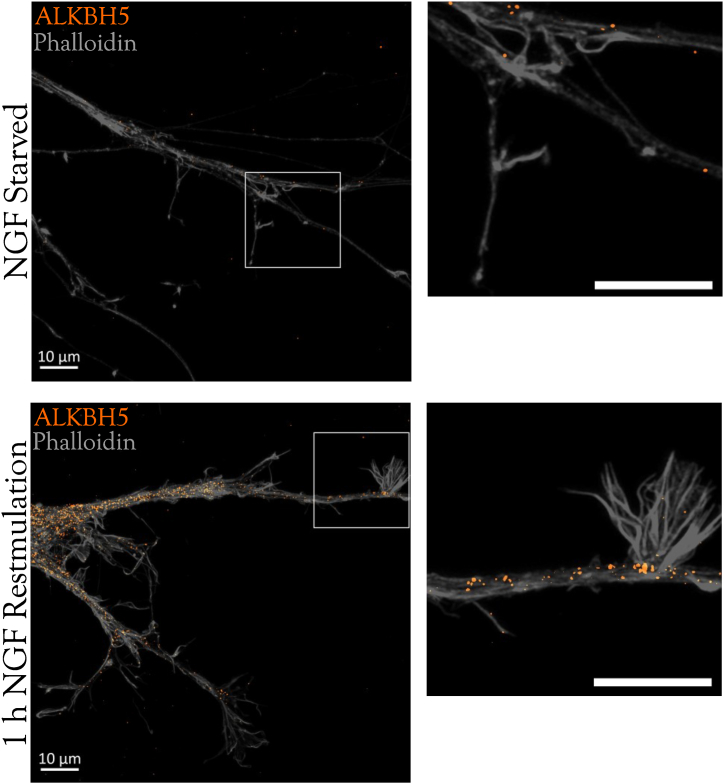
Sympathetic neuron explants were grown with NGF and after 5 days, either deprived of NGF for 18 h (top panels) or re-stimulated with NGF for 1 h (bottom panels). Axons were visualised with phalloidin staining (grey) and stained for the ALKBH5 demethylase (orange; Abcam ab195377).

All m^6^A readers mentioned above bind to the 3′ UTR of neuronal transcripts (Andreassi and Riccio, 2009). Thus, it is likely that FMRP, IGF2BPs, and HuD binding depends on m^6^A modifications of the target mRNAs, either through structural m^6^A switches or by forming protein complexes anchored by YTHDF proteins. Indeed, HuD transports and stabilises *Gap-43* and *Bdnf* transcripts (Yoo et al., 2013; Allen et al., 2013). Local protein synthesis of transcripts transported to dendrites and axons is essential for neurodevelopment and synaptic plasticity. In the 1990s, Frey & Morris described synaptic tagging as a mechanism by which, following initial synaptic stimulation, synapses are marked for further remodelling and become susceptible to long term potentiation (LTP), a protein-synthesis-dependent mechanism that increases synaptic strength (Frey and Morris, 1997; Redondo and Morris, 2010). m^6^A methylation of dendritic transcripts may contribute to synaptic tagging. Dendritic localisation of m^6^A readers for example, can either enhance mRNA translation or prompt transcript decay, depending on the synapse's state. Indeed, m^6^A-methylated mRNAs are transported to synapses and locally translated by YTHDF1 after induction of LTP (Merkurjev et al., 2018) and that YTHDF1 knockout mice have impaired late-LTP and memory consolidation (Shi et al., 2018). Similarly, m^6^A readers and m^6^A-mRNA have been detected in axons where they regulate mRNA transport and translation (Worpenberg et al., 2021). m^6^A erasers such as FTO and ALKBH5 also colocalise with m^6^A-mRNA in differentiated neuronal cell lines, mouse DRG sensory neuron axons, and rat SCG sympathetic neuron axons (Martinez De La Cruz et al., 2021; Yu et al., 2018) (Fig. 2).

Collectively, these data suggest that mRNA methylation may influence the binding, translation, and decay of localised transcripts. It is also clear that m^6^A methylation plays a key role in activity-dependent, temporal control of localised mRNA in neurons, although further studies are needed to reveal the additional mechanisms involved.

## 4 Significance of m^6^A mRNA modifications for human disorders

Given the widespread nature of m^6^A modifications, it is not surprising that changes of mRNA methylation have been involved in several human diseases. Alterations of m^6^A levels have been observed in a variety of human disorders, from obesity (Church et al., 2010) to type 2 diabetes (Xiao et al., 2016b), to infertility and cancer. For example, mutations of FTO and METTLs are often found in acute myeloid leukaemia (Weng et al., 2018) and glioblastoma (Cui et al., 2017b). Studies performed on various human cancers suggest that METTL3 activity is often dysregulated, perhaps indicating an additional therapeutic route for targeting neoplastic cells. For more detailed information, we refer the reader to a recent review in the role of m^6^A modified RNA and disease by Jiang et al. (Jiang et al., 2021).

## 5 Conclusions and outlook

Many aspects of the relationship between mRNA m^6^A modifications and metabolism remains unclear. Although several studies have described m^6^A mRNA, the accuracy and replicability of the data depends on the technique used. Therefore, the impact of m^6^A on mRNA metabolism in cases when only small quantities of tissue is available, such as neurons, needs to be investigated using more recent, high-sensitivity technology (Box 1 and Table 1). We are now beginning to understand how m^6^A modifications of the mRNA impacts on mRNA secondary structure and the binding of m^6^A readers and erasers. As m^6^A modifications and m^6^A-binding proteins are further investigated, biophysical properties like LLPS can now be studied in conjunction with more classical RNA-protein techniques, thereby providing essential information on the physical state associated with RNA modifications. Light-activated methods to control phase separation, such as optical tweezers (Bustamante et al., 2021) or optogenetic systems like OptoDroplets (Shin et al., 2017) may be used to induce phase-switches in different systems both *in vitro* and *in vivo*, allowing to study gene expression, m^6^A modifications and RNA binding to protein complexes. Data obtained from such studies could complement and validate *in silico* RNA-protein interaction predictions or RNA folding approaches (Wei et al., 2022; Sato et al., 2021). Together, these techniques will help elucidate the structural and physical changes undergone by m^6^A modified RNAs in healthy and diseased tissues.

Hundreds of RNA modifications are reported in the Modomics database of RNA modifications (Boccaletto et al., 2022), however the number detected on mRNAs is relatively small. During the past ten years, more modifications akin to m^6^A have been discovered. They include m^6^A_m_ and m^1^A, two modifications with a history and functions closely related to m^6^A (Mauer et al., 2016; Safra et al., 2017). Although m^6^A_m_ and m^1^A have not been thoroughly studied yet, it appears that they are less common than m^6^A (Li et al., 2017b; Sun et al., 2021). They also have functions similar to m^6^A, as they regulate mRNA stability and translation efficiency (Mauer et al., 2019; Wei et al., 2018). An exciting area of future research is centred on understanding how these modifications interact and/or compete with m^6^A to determine mRNA fate.

Finally, a limitation of our current knowledge is that only one m^6^A modification is studied at the time, and often at the 3′ UTR. This is despite the fact that m^6^A sites are also found within the coding sequence and the 5′ UTR, although less frequently (Meyer et al., 2012; Dominissini et al., 2012; Martinez De La Cruz et al., 2021; Flamand and Meyer, 2022). Given the higher number of m^6^A modifications per transcript in neurons (around 4–5 but can be up to 28) (Meyer et al., 2012; Dominissini et al., 2012; Martinez De La Cruz et al., 2021; Flamand and Meyer, 2022), it will be important to understand how multiple m^6^A modifications along a single mRNA affect RNA transport, translation, and decay, especially in dendrites and axons (Martinez De La Cruz et al., 2021). We now know that multi-modified mRNAs may influence LLPS, however how such changes affect accessibility of m^6^A-binding proteins to their target transcripts remains unclear. Are m^6^A modifications that induce structural changes found in clusters or distributed along a transcript? Do mRNA modifications always serve as protein-binding sites or in some cases, transcripts modifications primarily affect mRNA folding? It is possible that many answers lie in mRNA's structure, especially of the long and pliable 3′ UTR, where form and function may collide to finely tune gene expression.

BOX 1 **m6A sequencing technologies**

Over the last decade, there has been remarkable progress in epitranscriptomic sequencing technologies. One of the biggest barriers to the development of new and more efficient m^6^A sequencing techniques is the lack of chemical methods that reliably distinguish modified adenosine from unmodified (Grozhik et al., 2017a). For this reason, the sequencing methods of m^6^A initially focused on approaches based on antibody immunoprecipitation. However, in the last few years more chemical reaction-based methods have emerged. Initial methods for m^6^A sequencing were developed simultaneously in 2012 by two independent groups. m^6^A-seq (Dominissini et al., 2012) and meRIP-seq (Meyer et al., 2012) are methods in which total RNA (or poly-A tailed RNA from total RNA- 400 μg mRNA or 2.5 mg total RNA) is isolated and fragmented into ∼100 nt oligonucleotides followed by RNA immunoprecipitation with m^6^A antibodies (Meyer and Jaffrey, 2014b). The high-throughput RNA sequencing generates m^6^A peaks with a resolution ∼24 nt around the methylation site but it does not identify specific m^6^A residues (Dominissini et al., 2012). In 2013, Schwartz et al. increased the sensitivity of the method at a nearly single-nucleotide resolution (Schwartz et al., 2013). However, even though these sequencing methods generate m^6^A peaks, they are not sufficiently accurate to predict specific m^6^A residue on a transcriptome-wide level (Linder et al., 2015).

Methods that achieve higher resolution on transcriptome-wide m^6^A profiling are based and adapted from the original protocol of UV **C**ross-**L**inking and **I**mmuno**P**recipitation methodology named CLIP. In **P**hoto-crosslinking **A**ssisted m^6^A sequencing or PA-m^6^A-seq, an adaptation of **P**hoto**a**ctivatable-**R**ibonucleoside-Enhanced Cross linking and Immunoprecipitation (PAR-CLIP), 4-thiouridine (4SU) is incorporated into the mRNA (Li et al., 2016). Following immunoprecipitation with m^6^A antibody and UV crosslinking, the T to C transition allows the detection of the methylation sites (Chen et al., 2015) with a ∼23 nt resolution throughout the transcriptome. The **m**^6^A **i**ndividual nucleotide resolution cross-linking and immunoprecipitation (miCLIP) is an adaptation of the iCLIP methodology (Lee and Ule, 2018a, 2018b; Huppertz et al., 2014) based on specific mutational signatures induced by m^6^A antibodies. This allows the profiling of m^6^A and m^6^A_m_ residues at a single nucleotide resolution. The m^6^A antibodies are crosslinked with the RNA with UV irradiation to generate covalent bonds between the antibody and the fragmented RNA (Grozhik et al., 2017a). The covalent bonds introduce specific mutagenic signatures or truncations enabling the detection of m^6^A residues in the RNA (Hawley and Jaffrey, 1002). More sequencing approaches such as the m^6^A-LAIC-seq have been recently developed to quantify the stoichiometry of m^6^A modifications (Molinie et al., 2016).

Despite the rapid technology progress, the techniques available need improvements, given the large amount of input still required, the efficiency and sensitivity of the m^6^A antibody, and the cross-reactivity with other modifications. The Kouzarides lab developed a flexible and versatile method that detects RNA modifications from the DRS dataset in signal space Nanocompore (Leger et al., 2021). This new sequencing method allows direct sequencing from native RNA molecules (∼30 μg total RNA) and does not require the generation of a cDNA library. Most importantly, Nanocompore can identify multiple types of RNA modifications at a single molecule resolution. It is based on the comparison of the sample of interest with the reference sample devoid of specific modifications. The reference sample ideally derives from cells which do not express (knock-down or knock-out) the enzyme that catalyses the RNA modification (Leger et al., 2021). It should be noted however that the high accuracy of this method is achieved at the expense of sensitivity (Grozhik et al., 2017).

DART-seq or **D**eamination **A**djacent to **R**NA modification **T**argets **seq**uencing is an antibody-free RNA sequencing method (Wei et al., 2018) based on a targeted deamination strategy that uses the enzyme cytidine deaminase apolipoprotein B enzyme (APOBEC1) fused to m^6^A YTH domain to edit cytosine to uracil (C to U). When cells are transfected with APOBEC1-YTH C to U deamination is induced in sites adjacent to m^6^A residues. The most important innovation of this technique is the low amount of input RNA (10 ng) that can be used to map RNA modifications. All current methods of m^6^A mapping analyse data from cell populations. The low amount of RNA required for DART-seq allowed the development of a more advanced method for single-cell sequencing named the scDART-seq (Tegowski et al., 2022). With this sequencing technique, Tegowski et al. discovered a high heterogeneity among m^6^A methylome among single cells (Yao et al., 2022).

**R**NA **E**ndoribonuclease-**F**acilitated sequencing or m^6^A-REF-seq is an antibody-independent, high -throughput and single base m^6^A detection method that relies on MazF, an endoribonuclease, that recognizes the ACA motif and is sensitive to m^6^A (Zhang et al., 2019; Zhao et al., 2020). Adenosine methylated within the ACA motif cannot be cleaved by MazF, leaving the methylated (m^6^A)CA motif intact (Zhang et al., 2019). Although the technique can work with low amounts of RNA input (nanograms or even picograms), the main limitation is that the detection is limited to ACA motifs (Zhang et al., 2019). A similar sequencing method is the MAZTER-seq (Garcia-Campos et al., 2019) that although relying on the same principle uses a computational pipeline name MAZTER-MINE to identify the metylation sites. This method provides stoichiometry information but does not allow global mapping of m^6^A (Garcia-Campos et al., 2019).

m^6^A-label-seq is a recent metabolic labelling approach that uses the methionine analogue *Se* allyl-L-selenohomocysteine to substitute the methyl group on the enzyme S-adenosyl methionine (SAM) with the allyl (Shu et al., 2020). The presence of *Se* allyl- L-selenohomocysteine in cell cultures leads to N6- allyladenosine (a^6^A) modifications instead of m^6^A. The mRNA is isolated and fragmented from the treated with *Se* allyl- L-selenohomocysteine cells followed by antibody immunoprecipitation of a^6^A containing mRNA (Shu et al., 2022). The identification of the modified adenosines is based on iodination-induced misincorporation at the opposite site in the cDNA, during the library preparation. Even though this method is reliable, the low labelling yield, the lack of long labelling period, and the cellular stress induced by the methionine analogue will need improvement.

The FTO-assisted m^6^A selective chemical labelling method called m^6^A-SEAL is a chemical and antibody-free method (Wang et al., 2020) that combines two reactions: the FTO enzymatic oxidation of m^6^A and DTT-mediated thiol addition reaction. m^6^A-SEAL is a reliable and robust method, with a ∼200 nt resolution for transcriptome-wide detection of m^6^A. Depending on the reaction conditions, m^6^A-SEAL can distinguish m^6^A and cap m^6^A_m_ modifications, providing a great advantage when compared to the other methods (Wang et al., 2020).

One of the latest m^6^A technology is the m^6^**A**-**S**elective **A**llyl **C**hemical labelling and sequencing or m^6^A-SAC-seq. This method is based on the Dim1/KsgA dimethyltransferases, which transfer the methyl-group from SAM to adenosines, forming initially m^6^A and m^6^_2_A in a constitutive methylation reaction (Hu et al., 2022). It uses poly-A tailed RNA as input (∼30 ng) and provides m^6^A profiling at a single-base resolution with stoichiometry information. Although it prefers the GAC over AAC motif, it is quite accurate with a great potential for new biological discoveries.

## CRediT authorship contribution statement

**Braulio Martinez De La Cruz:** Conceptualization, Writing – original draft, Writing – review & editing. **Marousa Darsinou:** Conceptualization, Writing – original draft, Writing – review & editing. **Antonella Riccio:** Conceptualization, Supervision, Funding acquisition, Writing – original draft, Writing – review & editing.

## Data Availability

No data was used for the research described in the article.
